# Supplementary material for: ZNF143 mediates CTCF-bound promoter–enhancer loops required for murine hematopoietic stem and progenitor cell function
Source: Nat Commun. 2021 Jan 4;12:43. doi: 10.1038/s41467-020-20282-1 (PMC7782510; doi:10.1038/s41467-020-20282-1)
Supplement: Supplementary file 1 — Supplementary Information [file 41467_2020_20282_MOESM1_ESM.pdf]

Supplementary Figure 1

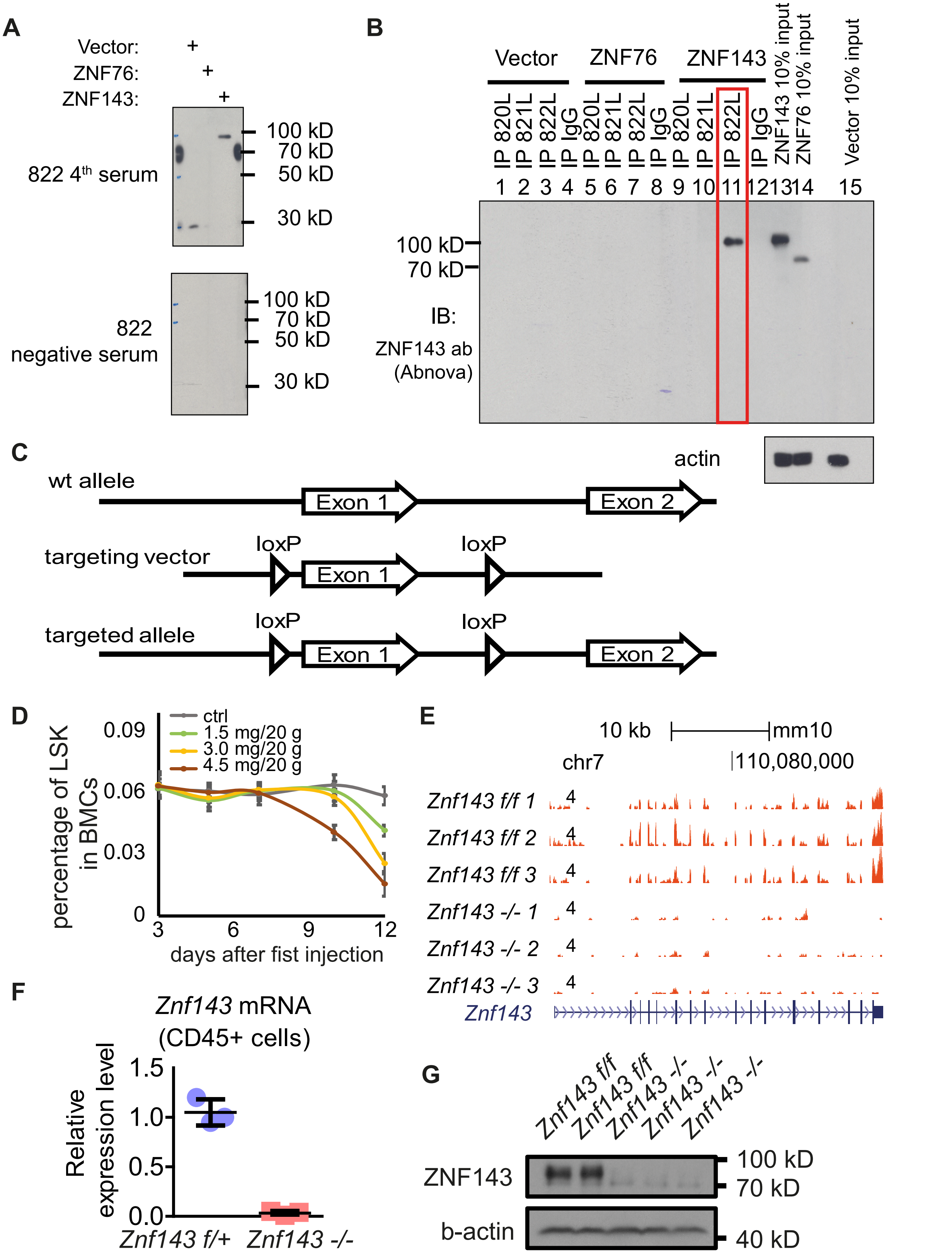

Supplementary Figure 1. Validation anti-ZNF143 rabbit serum and *Znf143* deletion in hematopoietic cells.

(**A**) Western-blot analysis to examine antibody specificity. The pPAC empty vector, pPAC-ZNF76, and pPAC-ZNF143 plasmids (expressing ZNF76 and ZNF143, respectively) were transfected into Schneider Drosophila S2 cells separately for 2 days. Equal amounts of cell lysate were loaded accordingly for Western-blot and incubated with different anti-ZNF143 rabbit serum as indicated. Negative serum stands for serum taken from the same rabbit before the first ZNF143 antigen immunization. The experiment has been repeated independently for three times with similar results. (**B**) IP to examine antibody specificity. The pPAC empty vector, pPAC-ZNF76, and pPAC-ZNF143 were overexpressed in S2 cells. Equal amounts of cell lysate were used for IP with different anti-ZNF143 rabbit serum or rabbit normal IgG. IP results were examined by Western-blot with commercial anti-ZNF143 mouse monoclonal antibody, which detects both ZNF76 and ZNF143. The experiment has been repeated independently for three times with similar results. (**C**) Cre-recombinase-mediated excision was designed to remove exon 1 of *Znf143* by inserting loxP sites before and after exon 1. (**D**) The curve describes the percentage changes of LSK in BMCs isolated from mice treated as indicated in *Methods*.
n = three biological replicates in each group. Values are presented as mean ± SD. (**E**) UCSC genome browser snapshot of RNA-seq result on *Znf143* locus. 3 pairs of littermates of *Znf143 f/f* x *Rosa26ERT2-Cre-* and *Znf143 f/f x Rosa26ERT2-Cre+* male mice were treated with middle dosage tamoxifen i.p injection for 10 days as in (D). RNA-seq was performed using the sorted HSLAM population (LSK+ CD150+ CD48-) was visualized on the UCSC genome browser. (**F**) CD45+ cells were sorted for RT-qPCR using fetal liver cells from littermates of *Znf143 f/+* x *Vav1-iCre-* and *Znf143 f/f* x *Vav1-iCre+* embryos at day E14.5. Relative *Znf143*

mRNA levels were normalized with *b-actin*. n = three biological replicates. Values are presented as mean ± SD. (**G**) *Znf143 f/f* x *Mx1-Cre-* and *Znf143 f/f* x *Mx1-Cre+* mice were injected with 300 µg poly I:C for three consecutive days. C-kit+ cells were sorted for Western-blot from BMCs 8 days after the first poly I:C injection. The experiment has been repeated independently for three times with similar results.

Supplementary Figure 2

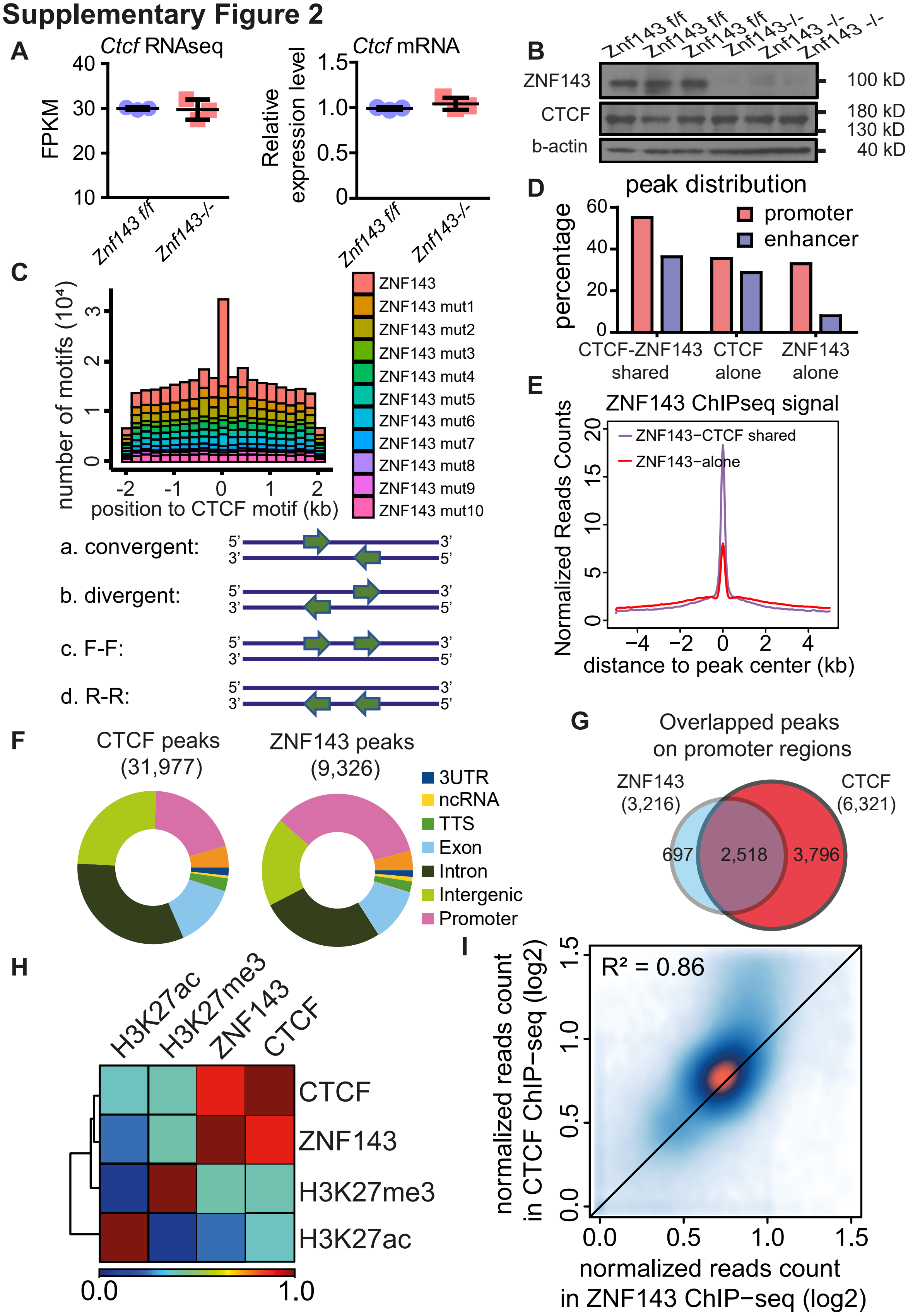

Supplementary Figure 2. CTCF and ZNF143 ChIP-seq peaks are strongly correlated.

(A) Left panel, the dot plot presents the RNA-seq signal (FPKM) of *Ctcf* in the HSLAM population (LSK+ CD150+ CD48-) from *Znf143 f/f* x *Rosa26ERT2-Cre-* (*Znf143 f/f*) and *Znf143 f/f* x *Rosa26ERT2-Cre+* (*Znf143 -/-*) mice treated with the middle dosage of tamoxifen for 10 days as described in Supplementary Figure 2B. n = three biological replicates. Values are presented as mean ± SD. Right panel, *Ctcf* RT-qPCR validation under same conditions. *Ctcf* mRNA is normalized to *β-actin*. n = three biological replicates. Values are presented as mean ± SD. **(B)** Western-blot of ZNF143 and CTCF proteins in HSPCs (c-kit+, lineage negative) sorted from mice treated under the same conditions as in (A). *β-actin* was used as the loading control. **(C)** Upper panel: Stacked histogram describing the distance from ZNF143 or ZNF143 mutated motifs (generated by *RSAT*, see STAR Methods for detail) to nearest CTCF motifs on the murine genome (mm10). The X axis represents the relative distance from ZNF143/ZNF143 mutated motifs to the CTCF motifs, while the Y axis represents the number of these motifs in each bin. Each bin size is 200 bp, and the coverage of the center bin is from -100 bp to +100 bp. Lower panel: Illustration of four possible patterns of motifs orientation between ZNF143 and the nearest CTCF motifs: 1) convergent on opposite strands (convergent); 2) divergent on opposite strands (divergent); 3) both on forward strand (F-F); 4) both on reverse strand (R-R). (**D**) Bar chart describing peak distribution of different subgroups of CTCF and ZNF143 peaks on promoter or enhancer regions. CTCF and ZNF143 ChIP-seq peaks detected in *Znf143 f/f* cells are sub-grouped into three groups (CTCF-ZNF143 shared, CTCF alone, or ZNF143 alone), and the percentage of peaks located on promoter or enhancer regions in each subgroup is presented. (**E**) The mean plot of ZNF143 ChIP-seq within ±4 kb from peak centers. ZNF143 peaks detected in *Znf143 f/f* cells were divided into 1) ZNF143-CTCF overlapped, and 2) ZNF143-alone. The X

axis represents the relative distance to the peak center, while the Y axis represents the mean of normalized read counts of the ZNF143 ChIP-seq signal in each group. **(F)** Annotation of ZNF143 and CTCF ChIP-seq peaks. HSPCs (c-kit+, lineage-) were sorted from wild type BMCs for ChIP-seq. **(G)** Venn diagram demonstrating peak overlaps between ZNF143 and CTCF ChIP-seq on promoter regions. **(H)** Cloud plot demonstrating the genome-wide correlation between ZNF143 and CTCF ChIP-seq signals. The X axis presents the normalized ZNF143 ChIP-seq signal, while the Y axis the normalized CTCF ChIP-seq signal. **(I)** Heatmap describes the correlation score among CTCF, ZNF143, the repressive histone mark (H3K27me3), and the active histone mark (H3K27ac) (Spearman correlation).

Supplementary Figure 3

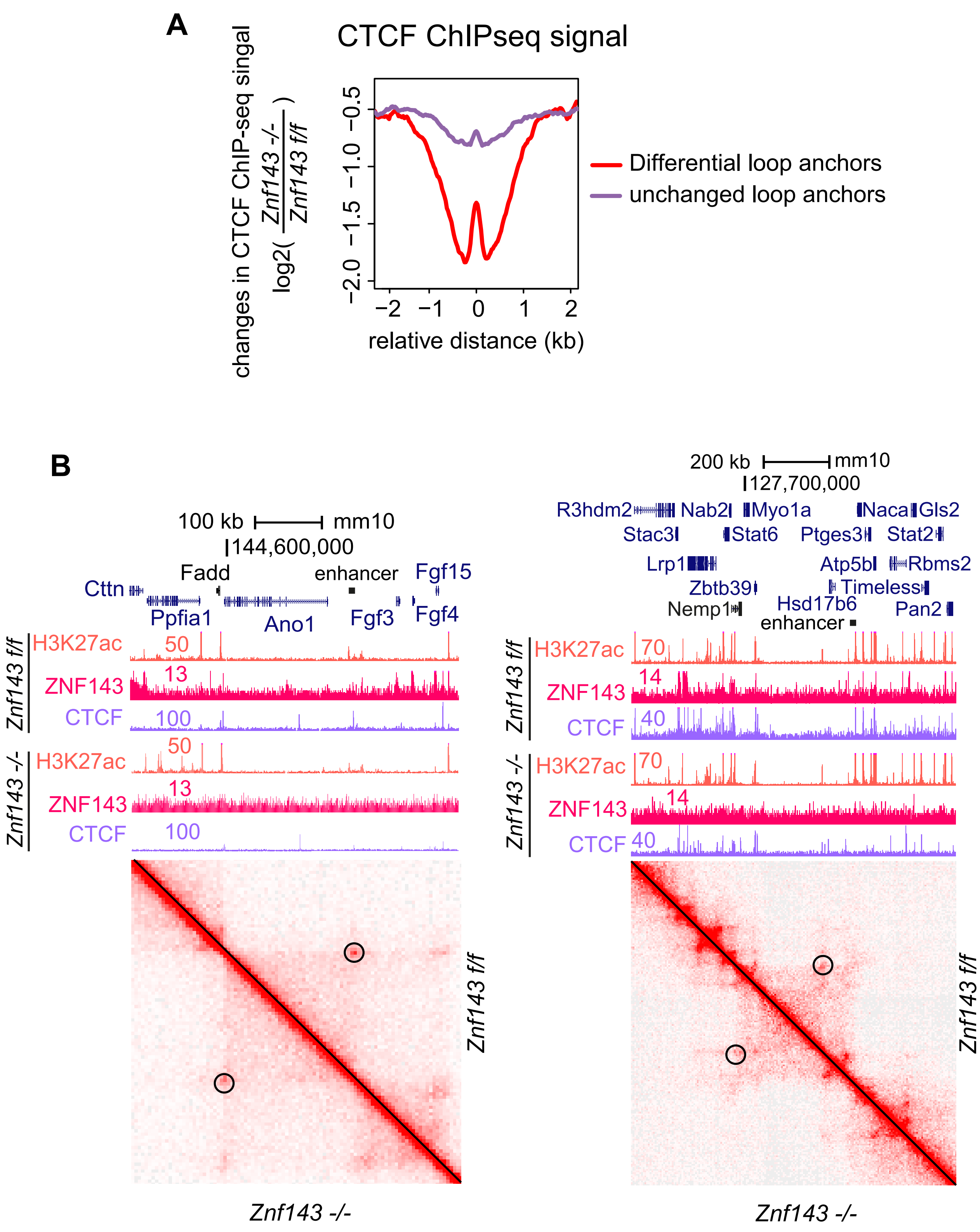

Supplementary Figure 3. Decreasing CTCF localization on ZNF143-related loop anchors after loss of ZNF143.

(**A**) Relative mean plot presents the average changes in the CTCF ChIP-seq signal within ±2 kb from CTCF motifs on loop anchors. Loops are divided into different groups as described in Figure 2A. The X axis represents relative distance to the CTCF motifs within loop anchors, while the Y axis represents the log2 fold changes of normalized read counts of CTCF ChIP-seq signals in each genomic region. n = two biological replicates. (**B**) Snapshots present HiC interaction frequencies, ChIP-seq profiles of ZNF143, CTCF, and H3K27ac, on *Fadd* locus (left panel) and *Nemp1* locus (right panel) in both *Znf143 f/f* and *Znf143 -/-* murine HSPCs. The balanced HiC two-dimensional contact matrix presents chromatin-chromatin interactions in *Znf143 f/f* (top-right part) and *Znf143 -/-* (bottom-left part) murine HSPCs. The color intensity presents interaction frequency, while black circles indicate the promoter-enhancer loop. ZNF143 and CTCF binding profile in *Znf143 f/f* or *Znf143 -/-* murine HSPCs are presented on top, while H3K27ac histone mark indicates an active chromatin status. The maximum values of Y axis are indicated in each track.

Supplementary Figure 4

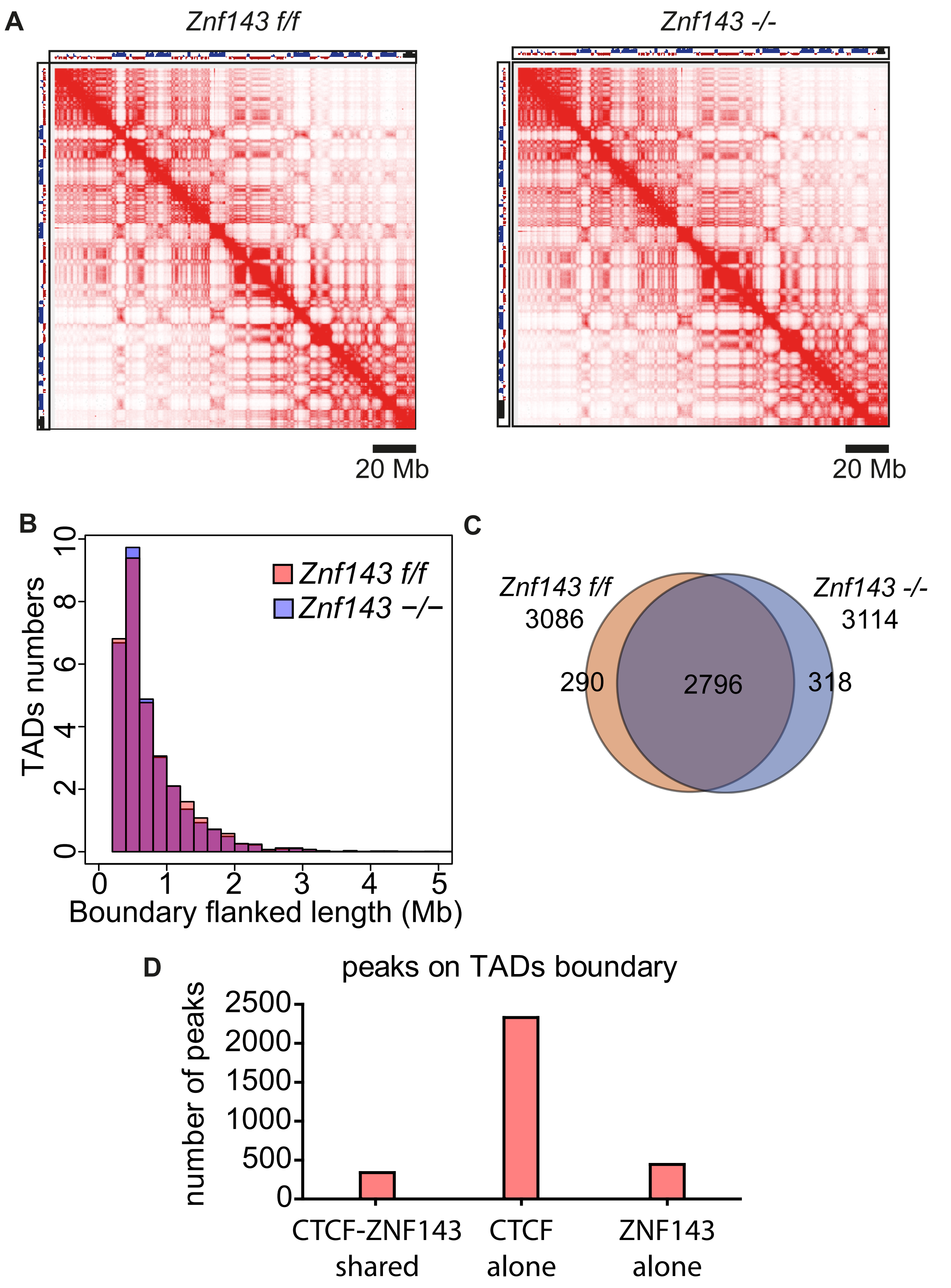

Supplementary Figure 4. Chromosome Compartments and TADs remain mainly unchanged after *Znf143* deletion.

(A) Comparison of chromosome compartments. Compartments on chromosome 1 (chr1) are demonstrated as an example in balanced contact matrices at 250 kb resolution. **(B)** Histogram describes the size distribution of TADs in *Znf143 f/f* (red) and *Znf143 -/-* (blue) cells. The X axis presents the boundary flanked lengths in each bin, while the Y axis the TAD numbers in each bin, with the red and blue bars representing the TAD numbers nearly overlapping. **(C)** Venn diagram describes overlapped TADs detected in *Znf143 f/f* and *Znf143 -/-* cells. (**D**) Bar chart presenting the number of peaks located on TADs boundaries. CTCF and ZNF143 peaks detected in *Znf143 f/f* cells were sub-grouped as described in Supplementary Figure 2D, and the number of peaks located on TADs boundaries in the different groups is presented.

Supplementary Figure 5

A

Vav1-iCre	+		-		total
ZNF143	f/+	f/f	f/+	f/f	
E14.5	13	15	12	14	54
E16.5	6	5	5	4	20
P1	14	0	11	7	32
Weaned	29	0	21	15	65

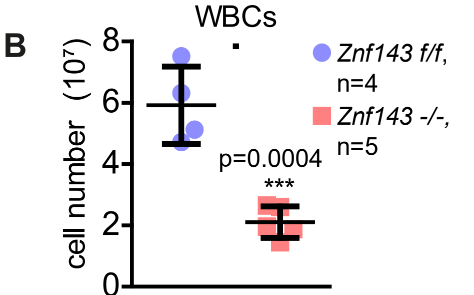

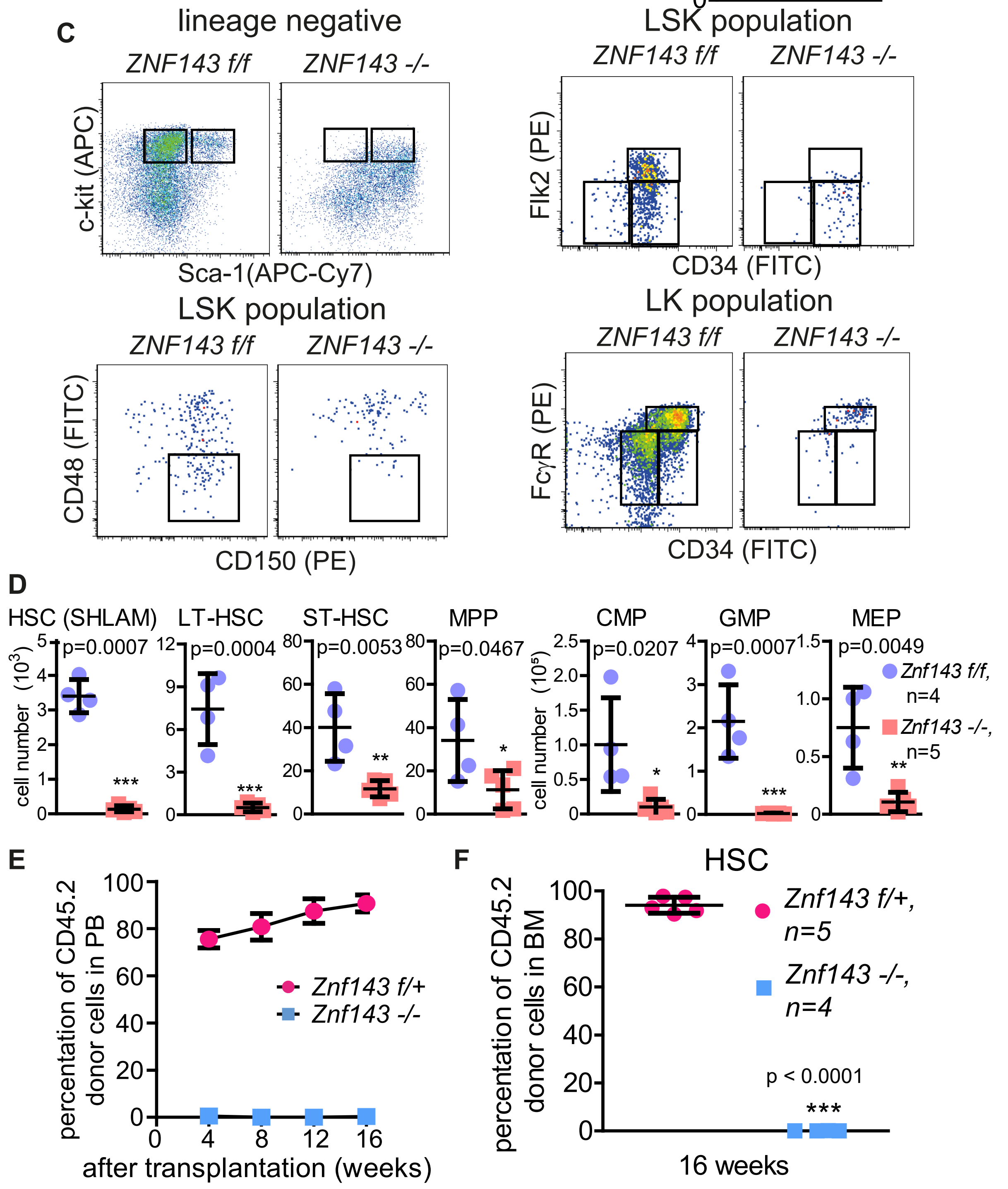

Supplementary Figure 5. Depletion of *Znf143* leads to a lethal phenotype at an embryonic stage and reduction of HSPC populations in adult mice.

(A) Summary of genotyping results of *Znf143* x *Vav1-iCre* embryos and mice. Genotyping of *Vav-iCre* and *Znf143* were performed at different embryonic periods and pup stages as indicated. (**B**) Dot plot describes absolute numbers of nucleated cells in wild type (*Znf143 f/f*) and *Znf143* deleted (*Znf143 -/-*) BMCs. **(C)** Dot plot of flow cytometry analysis of different HSPC populations in wild type (*Znf143 f/f*) and *Znf143* deleted (*Znf143 -/-*) BMCs. Littermates of *Znf143 f/f* x *Mx1-cre- and Znf143 f/f* x *Mx1-cre+* mice were injected with 300 µg poly I:C for three consecutive days. Eight days after the first injection, BMCs were harvested followed by flow cytometry examination. Surface markers were used to distinguish sub-populations LSK (Lin- Sca-1+ c-kit+) and LK (Lin- Sca-1- c-kit+). (**D**) Absolute numbers of HSPCs determined in (C). HSC: LSK CD150+ CD48-, LT-HSC: LSK CD34- Flk2-, ST-HSC: LSK CD34+ Flk2-, MPP: LSK CD34+ Flk2+, CMP: LK CD34+ FcγR-, GMP: LK CD34+ FcγR+, and MEP: LK CD34- FcγR-. Data in (B) and (D) was obtained from 4 of *Znf143 f/f* and 5 of *Znf143 -/-* littermates. Values are presented as mean ± SD. (**E**) Donor chimerism curve in peripheral blood (PB) describes the percentage engraftment after transplantation of wild type (*Znf143 f/+*) and *Znf143* deleted (*Znf143 -/-*) fetal liver cells. The X axis presents time after transplantation, while the Y axis percentage of CD45.2 donor cells in PB from recipients. *Znf143 f/+*: n = 5, *Znf143 -/-*: n = 4. Values are presented as mean ± SD. (**F**) Dot plots describe donor chimerism of HSCs in recipients 16 weeks after transplantation of wild type (*Znf143 f/+*) and *Znf143* deleted (*Znf143 -/-*) fetal liver cells. Each dot represents percentage of donor chimerism in one recipient mouse. *Znf143 f/+*: n = 5, *Znf143 -/-*: n = 4. Values are presented as mean ± SD. P values in (B), (D) and (F) are determined by two tailed unpaired t-test (*$p < 0.05$, **$p < 0.01$***$p < 0.001$).

Supplementary Figure 6

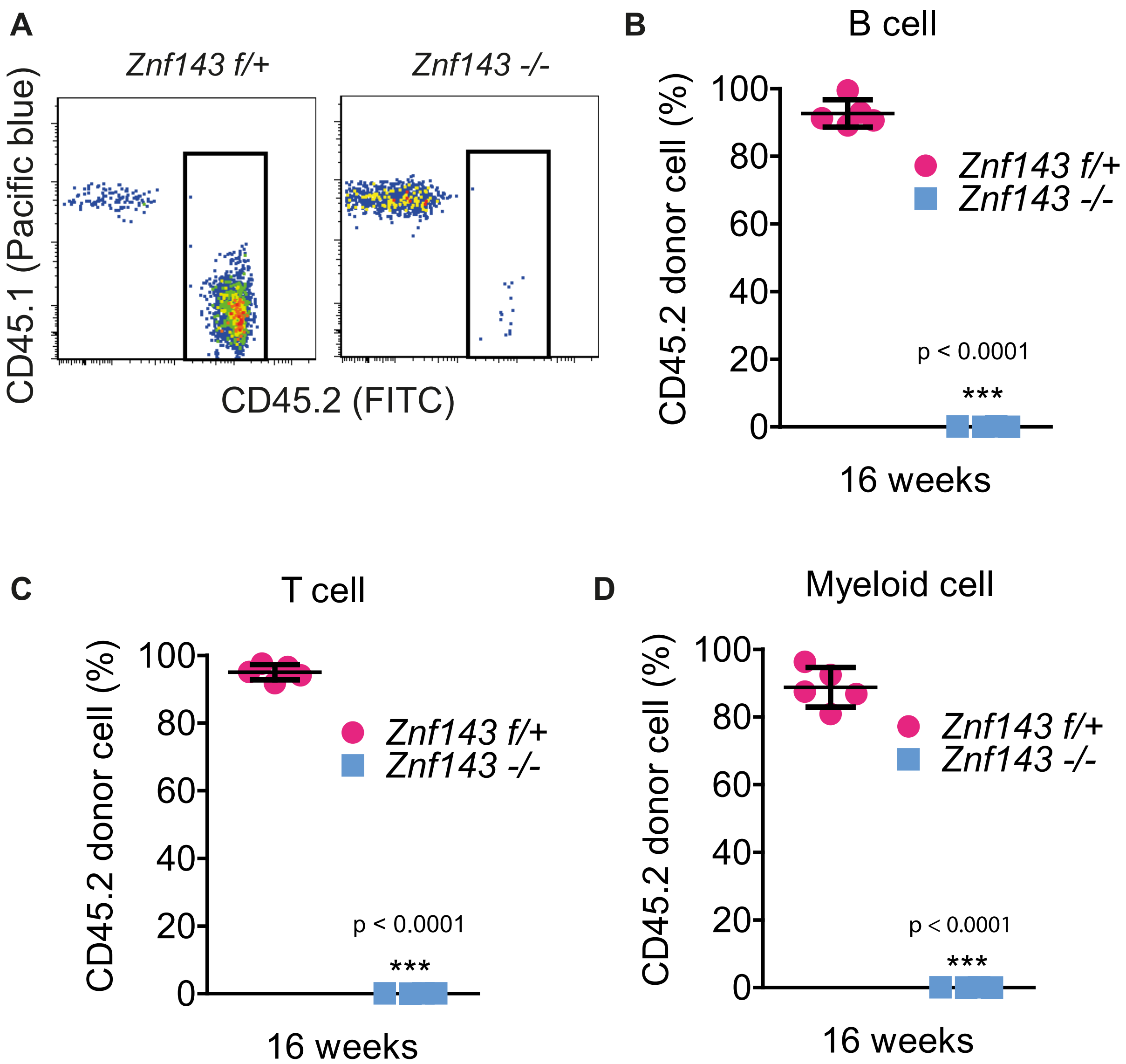

Supplementary Figure 6. Fetal liver HSPCs fail to reconstitute the hematopoietic system in transplantation after deletion of *Znf143*.

(**A**) Dot plot of flow cytometry demonstrating separation of donor-derived cells in peripheral blood from recipients. Cells derived from wild type (*Znf143 f/+*) or *Znf143* depleted (*Znf143 -/-*) fetal liver cells are indicated as the CD45.2 positive population, whereas competitor derived cells are indicated as the CD45.1 positive population. (**B-D**) Dot plots describe donor chimerism of

B cells (B), T cells (C), and myeloid cells (D) in recipients 16 weeks after transplantation. Each dot represents the percentage of donor chimerism in one recipient mouse (*Znf143 f/+*, n = 5; *Znf143 -/-*, n =4.). Values are presented as mean ± SD. P values in (B-D) are determined by a two tailed unpaired t-test (***$p < 0.001$).

Supplementary Figure 7

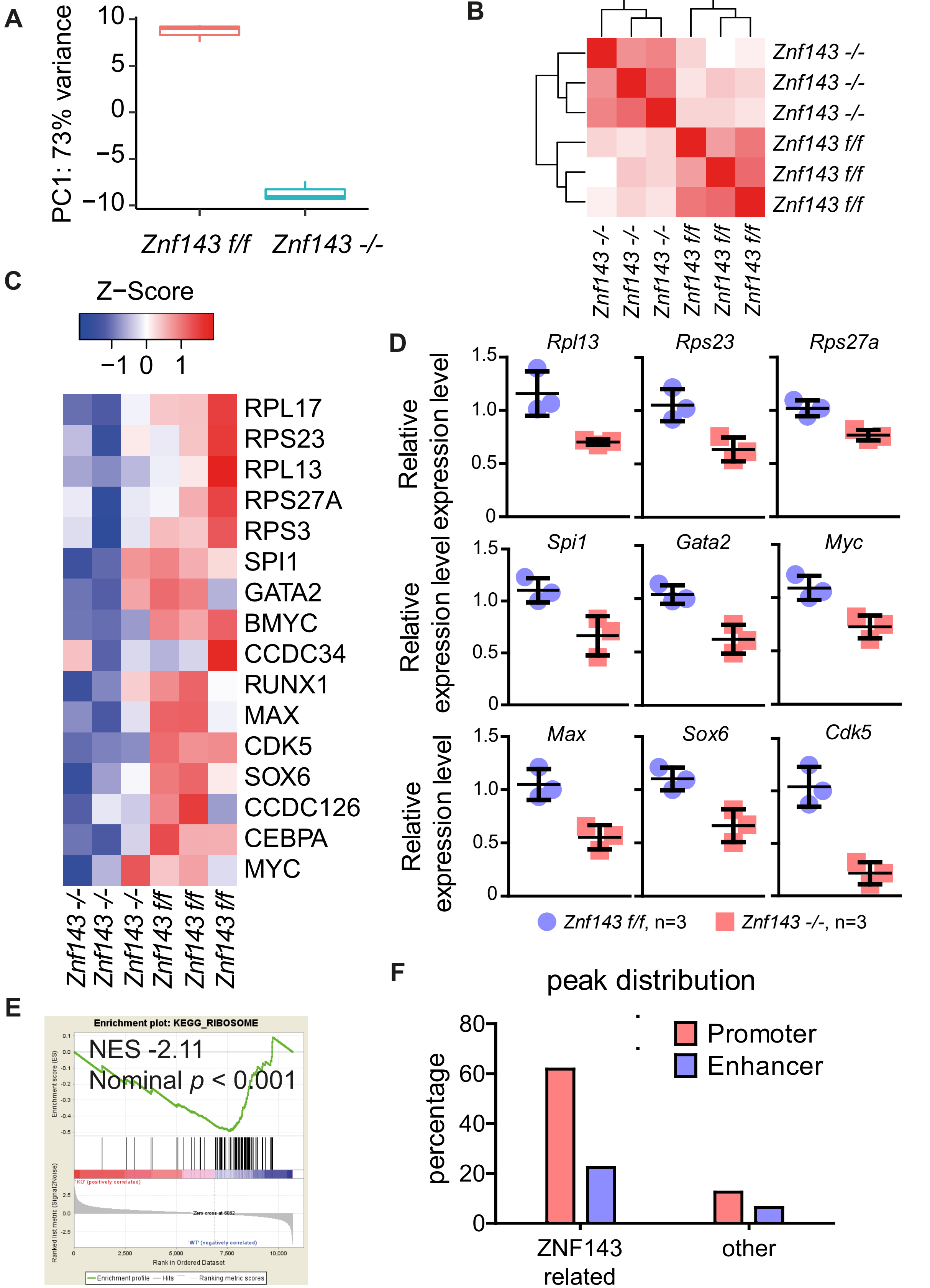

Supplementary Figure 7. Deletion of *Znf143* leads to changes of transcription profile.

(**A**) Box plot describes principal component analysis of RNA-seq data from wild type (*Znf143 f/f*) and *Znf143* depleted (*Znf143 -/-*) SLAM population (LSK+ CD150+ CD48-). Y axis presents first component value in each genotype. Boxplot represents median and the 25th and 75th percentiles of the distribution. The upper and lower whisker represents data no further than 1.5 times the IQR from 25th or 75th percentiles. (**B**) Heatmap describes clustering result of RNA-seq data in (A) based on unsupervised hierarchical cluster analysis. (**C**) Heatmap describes RNA-seq result of selected genes related to hematopoietic stem cell function. (**D**) RT-qPCR validation of RNA-seq result on certain genes in (C). Relative expression levels are normalized to *β-actin*. n = three biological replicates. Values are presented as mean ± SD. (**E**) Gene set enrichment analysis (GSEA) plot of Ribosome pathway. NES means normalized enrichment score. *Znf143 -/-* versus *Znf143 f/f* is presented. P value is determined as described previously[46]. (**F**) Bar chart describing peak distribution of different subgroups of CTCF peaks on promoter or enhancer regions. CTCF peaks are sub-grouped as described in Figure 5C, and the percentage of peaks located on promoter or enhancer regions in each subgroup is presented.

Supplementary Figure 8

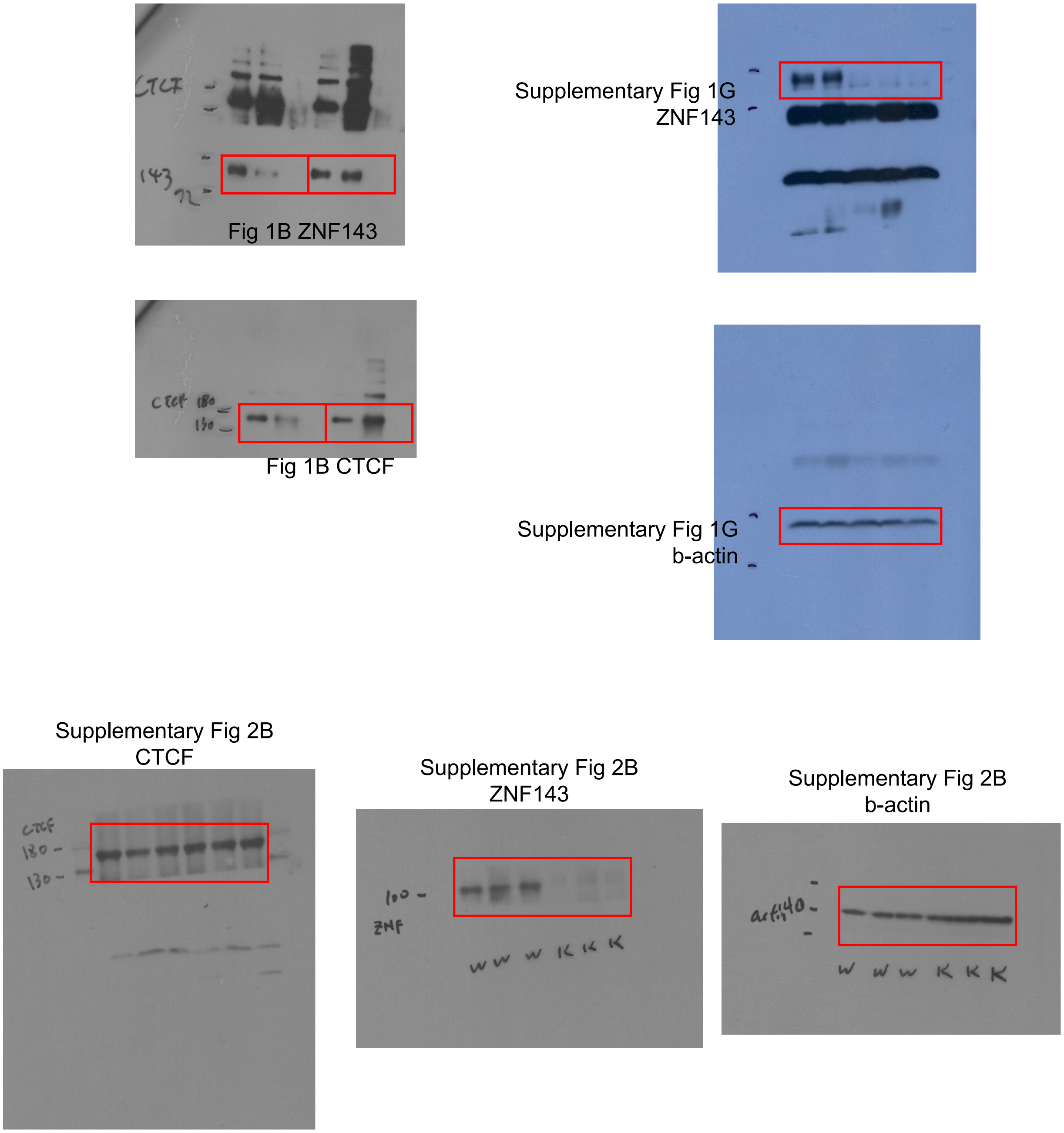

Supplementary Figure 8. Full scans of Western-blot.

Full scans of Western-blot of Figure 1B; Supplementary Figures 1G; 2B.

Supplementary Figure 9

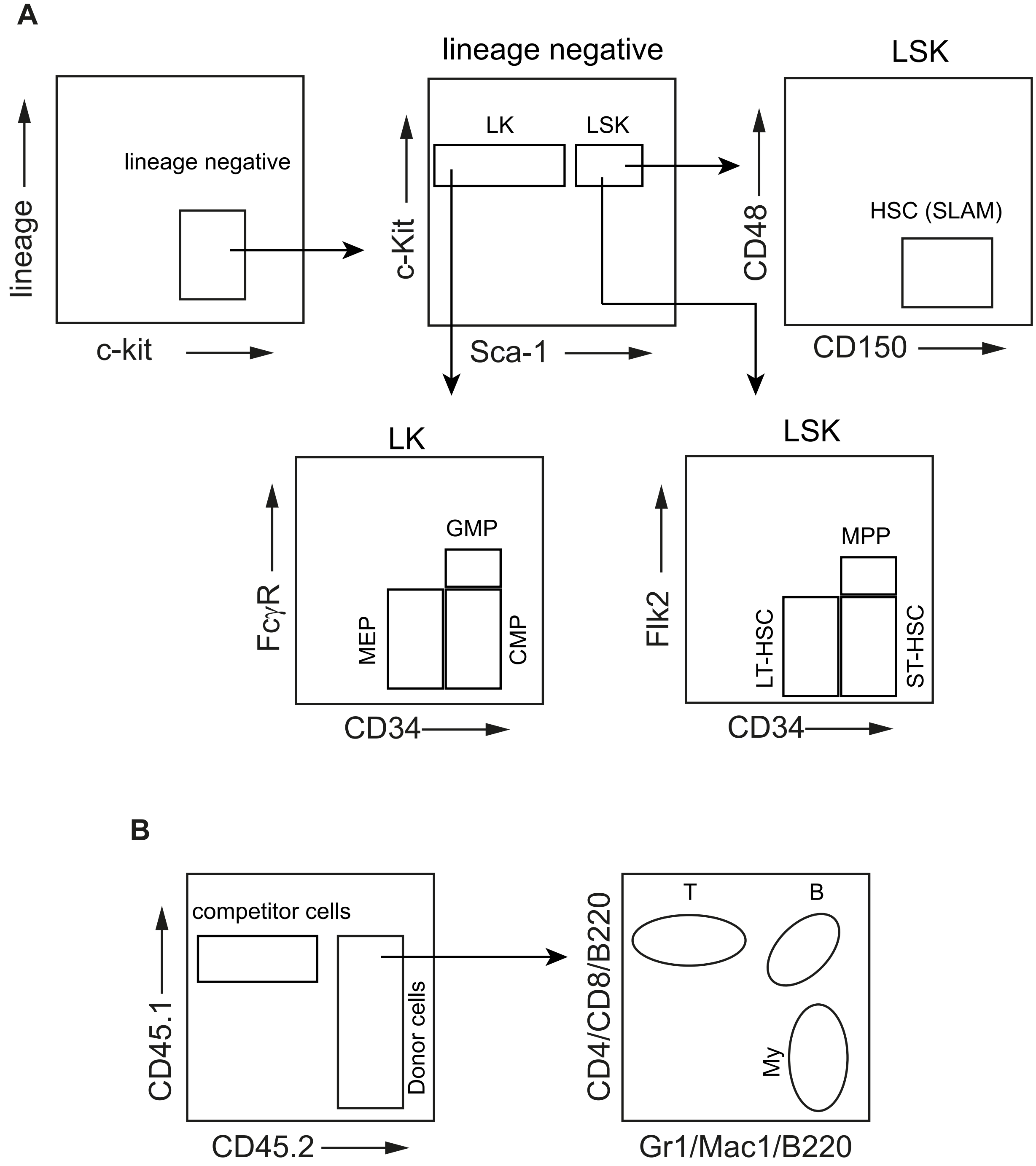

Supplementary Figure 9. Gating/sorting strategies for all flow cytometry and FACS.

Gating strategies to determine and/or sort (**A**) lineage negative, LSK, LK, HSC (SLAM),

LT-HSC, ST-HSC, MPP, CMP, GMP, and MEP population in fetal liver cells or BMCs; (**B**) donor cell, competitor cell; donor-cell-derived B-cell, T-cell, and myeloid cell population presented on Figures 4C-F, Supplementary Figures 5C-F, 6.
