# Supplementary material for: ZNF143 mediates CTCF-bound promoter–enhancer loops required for murine hematopoietic stem and progenitor cell function
Source: Nat Commun. 2021 Jan 4;12:43. doi: 10.1038/s41467-020-20282-1 (PMC7782510; doi:10.1038/s41467-020-20282-1)
Supplement: Supplementary file 3 — Description of Additional Supplementary Files [file 41467_2020_20282_MOESM3_ESM.pdf]

**Description of Additional Supplementary Files**

File Name: Supplementary Data 1
Description: CTCF peaks clustering by ZNF143

File Name: Supplementary Data 2
Description: CTCF and ZNF143 motifs located within 100bp

File Name: Supplementary Data 3
Description: Differential expressed gene list (P value is determined by Wald test)

File Name: Supplementary Data 4
Description: HiC loops list

File Name: Supplementary Data 5
Description: Annotation of ZNF143 affected loops

File Name: Supplementary Data 6
Description: CTCF motifs detected on loop anchors

File Name: Supplementary Data 7
Description: HiC detected TADs list

File Name: Supplementary Data 8
Description: List of Genes with strong ZNF143 association

File Name: Supplementary Data 9
Description: Staining strategy for different cell populations

File Name: Supplementary Data 10
Description: List of antibodies used in this study

File Name: Supplementary Data 11
Description: List of primers used in this study
