# Supplementary material for: ZNF143 mediates CTCF-bound promoter–enhancer loops required for murine hematopoietic stem and progenitor cell function
Source: Nat Commun. 2021 Jan 4;12:43. doi: 10.1038/s41467-020-20282-1 (PMC7782510; doi:10.1038/s41467-020-20282-1)
Supplement: Supplementary file 15 — Reporting Summary [file 41467_2020_20282_MOESM15_ESM.pdf]

nature research

Corresponding author(s): Daniel G. Tenen

Last updated by author(s): Oct 29, 2020

Reporting Summary

Nature Research wishes to improve the reproducibility of the work that we publish. This form provides structure for consistency and transparency in reporting. For further information on Nature Research policies, see Authors & Referees and the Editorial Policy Checklist.

Statistics

For all statistical analyses, confirm that the following items are present in the figure legend, table legend, main text, or Methods section.

n/a	Confirmed	
☐	☒	The exact sample size (*n*) for each experimental group/condition, given as a discrete number and unit of measurement
☐	☒	A statement on whether measurements were taken from distinct samples or whether the same sample was measured repeatedly
☐	☒	The statistical test(s) used AND whether they are one- or two-sided<br>*Only common tests should be described solely by name; describe more complex techniques in the Methods section.*
☒	☐	A description of all covariates tested
☒	☐	A description of any assumptions or corrections, such as tests of normality and adjustment for multiple comparisons
☐	☒	A full description of the statistical parameters including central tendency (e.g. means) or other basic estimates (e.g. regression coefficient) AND variation (e.g. standard deviation) or associated estimates of uncertainty (e.g. confidence intervals)
☐	☒	For null hypothesis testing, the test statistic (e.g. *F*, *t*, *r*) with confidence intervals, effect sizes, degrees of freedom and *P* value noted<br>*Give P values as exact values whenever suitable.*
☒	☐	For Bayesian analysis, information on the choice of priors and Markov chain Monte Carlo settings
☒	☐	For hierarchical and complex designs, identification of the appropriate level for tests and full reporting of outcomes
☒	☐	Estimates of effect sizes (e.g. Cohen's *d*, Pearson's *r*), indicating how they were calculated

Our web collection on statistics for biologists contains articles on many of the points above.

Software and code

Policy information about availability of computer code

Data collection	Data collection was done with BD FACSDiva software (v8.0.1) for the flow cytometry experiment.<br>Illumina bcl2fastq2 Conversion Software (v2.20) was used for Next Generation Sequencing library demultiplexing.
Data analysis	R (v3.5.1), Rstudio (v1.1.456), ggplot (3.2.1), Fastqc (v0.11.5), STAR (v2.5.1b_modified), TrimGalore (v0.6.0), FeatureCounts (v1.5.2), DESeq2 (v1.22.2), GSEA (v4.0.3), IGV (v2.3.74), bedtools (v2.25.0), bedGraphToBigWig (v4), bowtie2 (v2.2.9), Samtools (v1.9), MACS2 (v2.1.1.20160309), HOMER (v4.10.3), RSAT (Sep 24 04:31:43 2020), deeptools (v3.1.1), ngsplot (v2.61), Juicer tools (v1.9.9_jcuda.0.8), Juicebox (1.11.08), HiC-Pro (2.11.3), FlowJo (v7.5) and GraphPad Prism (v5.01).<br>Custom code is available upon reasonable request.

For manuscripts utilizing custom algorithms or software that are central to the research but not yet described in published literature, software must be made available to editors/reviewers. We strongly encourage code deposition in a community repository (e.g. GitHub). See the Nature Research guidelines for submitting code & software for further information.

Data

Policy information about availability of data

All manuscripts must include a data availability statement. This statement should provide the following information, where applicable:

- Accession codes, unique identifiers, or web links for publicly available datasets
- A list of figures that have associated raw data
- A description of any restrictions on data availability

The ChIP-seq, RNA-seq, and HiC data that support the findings of this study have been deposited in GEO with the GEO accession number GSE144712 [https://www.ncbi.nlm.nih.gov/geo/query/acc.cgi?acc=GSE144712]. Biological material used in this study can be obtained from the authors upon request. The authors declare that all other data supporting the findings of this study are available within the paper and its supplementary information files. Source data are provided with this paper.

Field-specific reporting

Please select the one below that is the best fit for your research. If you are not sure, read the appropriate sections before making your selection.

☒ Life sciences ☐ Behavioural & social sciences ☐ Ecological, evolutionary & environmental sciences

For a reference copy of the document with all sections, see nature.com/documents/nr-reporting-summary-flat.pdf

Life sciences study design

All studies must disclose on these points even when the disclosure is negative.

Sample size	No statistical methods were used to predetermine sample sizes. Sample sizes were chosen in order to be able to perform statistical analyses, as is standard in the field (doi: 10.1182/blood.2019001279)
Data exclusions	No data were excluded from the analyses.
Replication	To verify the reproducibility of our findings, experiments were performed using at least three biological replicates, unless clearly stated otherwise in the figure legends. All attempts at replication were successful.
Randomization	Match randomization, according to age and gender, was applied to allocate animals to control or experimental groups.
Blinding	No blinding was applied for in vivo mice experiments and rest experiments during data collection as almost all data are quantitative and not easily subjected to investigator bias.

Reporting for specific materials, systems and methods

We require information from authors about some types of materials, experimental systems and methods used in many studies. Here, indicate whether each material, system or method listed is relevant to your study. If you are not sure if a list item applies to your research, read the appropriate section before selecting a response.

Materials & experimental systems

n/a	Involved in the study	
☐	☒	Antibodies
☐	☒	Eukaryotic cell lines
☒	☐	Palaeontology
☐	☒	Animals and other organisms
☒	☐	Human research participants
☒	☐	Clinical data

Methods

n/a	Involved in the study	
☐	☒	ChIP-seq
☐	☒	Flow cytometry
☒	☐	MRI-based neuroimaging

Antibodies

Antibodies used

antibodies for FACS
Ly-6G/Ly-6C Monoclonal Antibody (RB6-8C5) PE-Cyanine7 Cat # 25-5931-82 eBioscience Lot. E07648-1634 Clone: RB6-8C5
CD11b Monoclonal Antibody (M1/70) PE-Cyanine7 Cat # 25-0112-82 eBioscience Lot. 4289817 Clone: M1/70
CD3e Monoclonal Antibody (145-2C11) PE-Cyanine7 Cat # 25-0031-82 eBioscience Lot. 4293485 Clone: 145-2C11
anti-mouse CD4 PE-Cyanine7 Cat # 25-0041-82 eBioscience Lot. E07501-1634 Clone: GK1.5
CD8a Monoclonal Antibody (53-6.7) PE-Cyanine7 Cat # 25-0081-82 eBioscience Lot. E07510-1635 Clone: 53-6.7
CD45R (B220) Monoclonal Antibody (RA3-6B2) PE-Cyanine7 Cat # 25 0452-82 eBioscience Lot. E07569-1636 Clone: RA3-6B2
CD19 Monoclonal Antibody (eBio1D3 (1D3)) PE-Cyanine7 Cat # 25 0193 82 eBioscience Lot. E07526-1635 Clone: eBio1D3
TER-119 Monoclonal Antibody (TER-119) PE-Cyanine7 Cat # 25-5921-82 eBioscience Lot. E07646-1634 Clone: TER-119
CD117 (c-Kit) Monoclonal Antibody (2B8) APC Cat # 17-1171-82 eBioscience Lot. 4277766 Clone: 2B8
APC/Cy7 anti-mouse Ly-6A/E (Sca-1) Antibody Cat # 108126 BioLegend Lot. B214144 Clone: D7
PE anti-mouse CD150 (SLAM) Antibody Cat # 115904 BioLegend Lot. B127947 Clone: TC15-12F12.2
FITC anti-mouse CD48 Antibody Cat # 103404 BioLegend Lot. E00337-1630 Clone: HM48-1
anti-mouse CD34 FITC Cat # 11-0341-82 eBioscience Lot. 4276888 Clone: RAM34
PE Rat anti-mouse CD16/CD32 Cat # 553145 BD Biosciences Lot. 02479 Clone: 2.4G2
Anti-mouse CD135 (Flt-3) PE Cat # 12-1351-82 eBioscience Lot. E01494-1630 Clone: A2F10
Biotin anti-mouse Ly-6G/Ly-6C (Gr-1) Antibody Cat # 108404 BioLegend Lot. B171592 Clone: RB6-8C5
CD11b Monoclonal Antibody (M1/70) Biotin Cat # 13-0112-82 eBioscience Lot. E02411-1631 Clone: M1/70
Biotin anti-mouse CD3ε Antibody Cat # 100304 BioLegend Lot. B171606 Clone: 145-2C11
Biotin anti-mouse CD8a Antibody Cat # 100704 BioLegend Lot. B180225 Clone: 53-6.7
Biotin anti-mouse/human CD45R/B220 Antibody Cat # 103204 BioLegend Lot. B178627 Clone: RA3-6B2
Biotin anti-mouse CD19 Antibody Cat # 115504 BioLegend Lot. B192314 Clone: 6D5
Biotin anti-mouse TER-119/Erythroid Cells Antibody Cat # 116204 BioLegend Lot. B203465 Clone: TER-119

October 2018

CD45.1 Monoclonal Antibody (A20) eFluor 450 Cat # 48-0453-82 eBioscience Lot. E08504-1630 Clone: A20
FITC Mouse Anti-Mouse CD45.2 Clone 104 Cat # 553772 BD Biosciences Lot. 23167 Clone: 104
CD45R (B220) Monoclonal Antibody (RA3-6B2) APC Cat # 17-0452-82 eBioscience Lot. E07151-1632 Clone: RA3-6B2
APC anti-mouse Ly-6G/Ly-6C (Gr-1) Antibody Cat # 108412 BioLegend Lot. B166444 Clone: RB6-8C5
CD11b Monoclonal Antibody (M1/70) APC Cat # 17-0112-82 eBioscience Lot. E07073-1633 Clone: M1/70
Streptavidin MicroBeads Cat # 130-048-101 Miltenyi Biotec Lot. 5190628108
CD117 MicroBeads mouse Cat # 130-091-224 Miltenyi Biotec Lot. 5191010027
antibodies for ChIP and Western-blot
Anti-ZNF143 rabbit serum generated in our lab
Anti-CTCF antibody Cat# 07-729 Millipore Lot. 2757161
Tri-Methyl-Histone H3 (Lys27) Cat# 9733BC Cell Signaling Technology Lot. 8 Clone: C36B11
Rabbit polyclonal to Histone H3 (acetyl K27) - ChIP Grade Cat# ab4729 Abcam Lot. GR261979-1
Anti-ZNF143 mouse monoclonal (M01), clone 2B4 Cat# H00007702-M01 Abnova clone: 2B4
Anti-b-actin HRP conjugated Cat# sc-47778 HRP Santa Cruz Lot. J2915 Clone: C4

Validation

https://www.thermofisher.com/antibody/product/Ly-6G-Ly-6C-Antibody-clone-RB6-8C5-Monoclonal/25-5931-82
https://www.thermofisher.com/antibody/product/CD11b-Antibody-clone-M1-70-Monoclonal/25-0112-82
https://www.thermofisher.com/antibody/product/CD3e-Antibody-clone-145-2C11-Monoclonal/25-0031-82
https://www.thermofisher.com/antibody/product/CD8a-Antibody-clone-53-6-7-Monoclonal/25-0081-82
https://www.thermofisher.com/antibody/product/CD4-Antibody-clone-GK1-5-Monoclonal/25-0041-82
https://www.thermofisher.com/antibody/product/CD45R-B220-Antibody-clone-RA3-6B2-Monoclonal/25-0452-82
https://www.thermofisher.com/antibody/product/CD19-Antibody-clone-eBio1D3-1D3-Monoclonal/25-0193-82
https://www.thermofisher.com/antibody/product/TER-119-Antibody-clone-TER-119-Monoclonal/25-5921-82
https://www.thermofisher.com/antibody/product/CD117-c-Kit-Antibody-clone-2B8-Monoclonal/17-1171-82
https://www.biolegend.com/en-us/search-results/apc-cy7-anti-mouse-ly-6a-e-sca-1-antibody-6752
https://www.biolegend.com/en-us/products/pe-anti-mouse-cd150-slam-antibody-1369
https://www.biolegend.com/en-us/products/fitc-anti-mouse-cd48-antibody-291
https://www.thermofisher.com/antibody/product/CD34-Antibody-clone-RAM34-Monoclonal/11-0341-82
https://www.bdbiosciences.com/us/applications/research/b-cell-research/surface-markers/mouse/pe-rat-anti-mouse-cd16cd32-24g2/p/553145
https://www.thermofisher.com/antibody/product/CD135-Flt3-Antibody-clone-A2F10-Monoclonal/12-1351-82
https://www.biolegend.com/en-us/products/biotin-anti-mouse-ly-6g-ly-6c-gr-1-antibody-457
https://www.thermofisher.com/antibody/product/CD11b-Antibody-clone-M1-70-Monoclonal/13-0112-82
https://www.biolegend.com/en-us/products/biotin-anti-mouse-cd3epsilon-antibody-22
https://www.biolegend.com/en-us/products/biotin-anti-mouse-cd8a-antibody-152
https://www.biolegend.com/en-us/products/biotin-anti-mouse-human-cd45r-b220-antibody-444
https://www.biolegend.com/en-us/products/biotin-anti-mouse-cd19-antibody-1527
https://www.biolegend.com/en-us/products/biotin-anti-mouse-ter-119-erythroid-cells-antibody-1864
https://www.thermofisher.com/antibody/product/CD45-1-Antibody-clone-A20-Monoclonal/48-0453-82
https://www.bdbiosciences.com/sg/applications/research/stem-cell-research/cancer-research/mouse/fitc-mouse-anti-mouse-cd452-104/p/553772
https://www.thermofisher.com/antibody/product/CD45R-B220-Antibody-clone-RA3-6B2-Monoclonal/17-0452-82
https://www.biolegend.com/en-us/products/apc-anti-mouse-ly-6g-ly-6c-gr-1-antibody-456
https://www.thermofisher.com/antibody/product/CD11b-Antibody-clone-M1-70-Monoclonal/17-0112-82
https://www.miltenyibiotec.com/SG-en/products/macs-cell-separation/cell-separation-reagents/microbeads-and-isolation-kits/any-cell-type/streptavidin-microbeads.html#130-048-101
https://www.miltenyibiotec.com/SG-en/products/macs-cell-separation/cell-separation-reagents/microbeads-and-isolation-kits/hematopoietic-stem-cells/cd117-microbeads-mouse.html#130-091-224
https://www.merckmillipore.com/SG/en/product/Anti-CTCF-Antibody,MM_NF-07-729?ReferrerURL=https%3A%2F%2Fwww.google.com%2F
https://www.cellsignal.com/products/primary-antibodies/tri-methyl-histone-h3-lys27-c36b11-rabbit-mab/9733
https://www.abcam.com/histone-h3-acetyl-k27-antibody-chip-grade-ab4729.html
https://www.novusbio.com/products/znf143-antibody-2b4_h00007702-m01
https://www.scbt.com/p/beta-actin-antibody-c4

Eukaryotic cell lines

Policy information about cell lines

Cell line source(s)

Drosophila cell line Schneider2 (S2) was purchased from ATCC

Authentication

All cell lines have been authenticated by short tandem repeat analysis.

Mycoplasma contamination

All cell lines tested negative for mycoplasma.

Commonly misidentified lines
(See ICLAC register)

No commonly misidentified cell lines were used.

Animals and other organisms

Policy information about studies involving animals; ARRIVE guidelines recommended for reporting animal research

Laboratory animals

8-12 weeks old male C57BL/6J, Mx1-Cre, and Vav1-iCre mice were obtained from The Jackson Laboratory; Rosa26ERT2-Cre mice

Laboratory animals	were obtained from Yoshiaki Ito. Znf143 flox mice were generated by our lab. Znf143 f/f female mice (12-14 weeks old) were used for time mating; Znf143 f/f x Mx1 Cre+ (8-12 weeks old, male), Znf143 f/f; Mx1 Cre- (8-12 weeks old, male), Znf143 f/f x Rosa26ERT2 Cre+ (8-12 weeks old, male) Znf143 f/f x Rosa26ERT2 Cre- mice (8-12 weeks old, male) mice were used for Znf143 depletion as described in Methods.
Wild animals	The study did not involve wild animals.
Field-collected samples	The study did not involve samples collected from the field.
Ethics oversight	All animal experiments were performed with approval from the Institutional Animal Care and Use Committee of National University of Singapore

Note that full information on the approval of the study protocol must also be provided in the manuscript.

ChIP-seq

Data deposition

☒ Confirm that both raw and final processed data have been deposited in a public database such as GEO.

☒ Confirm that you have deposited or provided access to graph files (e.g. BED files) for the called peaks.

Data access links<br>*May remain private before publication.*	https://www.ncbi.nlm.nih.gov/geo/query/acc.cgi?acc=GSE144712<br>token: wtajigsiznmbtut
Files in database submission	1-pos_R1.fastq.gz<br>1-pos_R2.fastq.gz<br>2-pos_R1.fastq.gz<br>2-pos_R2.fastq.gz<br>3-pos_R1.fastq.gz<br>3-pos_R2.fastq.gz<br>4-neg_R1.fastq.gz<br>4-neg_R2.fastq.gz<br>5-neg_R1.fastq.gz<br>5-neg_R2.fastq.gz<br>6-neg_R1.fastq.gz<br>6-neg_R2.fastq.gz<br>KO_input_R1.fastq.gz<br>KO_input_R2.fastq.gz<br>KO-CTCF-ckit_R1.fastq.gz<br>KO-CTCF-ckit_R2.fastq.gz<br>KO-H3K27me3-ckit_R1.fastq.gz<br>KO-H3K27me3-ckit_R2.fastq.gz<br>KO-H3K27ac-ckit_R1.fastq.gz<br>KO-H3K27ac-ckit_R2.fastq.gz<br>KO-ZNF143_R1.fastq.gz<br>KO-ZNF143_R2.fastq.gz<br>WT-ZNF143_R1.fastq.gz<br>WT-ZNF143_R2.fastq.gz<br>WT-H3K27me3_R1.fastq.gz<br>WT-H3K27me3_R2.fastq.gz<br>WT_SGL01927_H2WG2BBXX_L7_1.fq.gz<br>WT_SGL01927_H2WG2BBXX_L7_2.fq.gz<br>WT_SGL01927_H2WG2BBXX_L8_1.fq.gz<br>WT_SGL01927_H2WG2BBXX_L8_2.fq.gz<br>KO_SGL01928_H2WFNBBXX_L1_1.fq.gz<br>KO_SGL01928_H2WFNBBXX_L1_2.fq.gz<br>KO_SGL01928_H2WFNBBXX_L2_1.fq.gz<br>KO_SGL01928_H2WFNBBXX_L2_2.fq.gz<br>WT-CTCF-ckit_R1.fastq.gz<br>WT-CTCF-ckit_R2.fastq.gz<br>WT-H3K27ac-ckit_R1.fastq.gz<br>WT-H3K27ac-ckit_R2.fastq.gz<br>WT_input_R1.fastq.gz<br>WT_input_R2.fastq.gz<br>KO-CTCF-ckit_2_R1.fastq.gz<br>KO-CTCF-ckit_2_R2.fastq.gz<br>KO-H3K27ac-ckit_2_R1.fastq.gz<br>KO-H3K27ac-ckit_2_R2.fastq.gz<br>KO-H3K27me3-ckit_2_R1.fastq.gz<br>KO-H3K27me3-ckit_2_R2.fastq.gz<br>WT-CTCF-ckit_2_R1.fastq.gz<br>WT-CTCF-ckit_2_R2.fastq.gz<br>WT-H3K27ac-ckit_2_R1.fastq.gz

WT-H3K27ac-ckit_2_R2.fastq.gz
WT-H3K27me3-ckit_2_R1.fastq.gz
WT-H3K27me3-ckit_2_R2.fastq.gz
WT-ZNF143-ckit_2_R1.fastq.gz
WT-ZNF143-ckit_2_R2.fastq.gz
KO_input_mm10.bowtie2.rd.bw
KO-CTCF-ckit_mm10.bowtie2.rd.bw
KO-H3K27me3-ckit_mm10.bowtie2.rd.bw
KO-H3K27ac-ckit_mm10.bowtie2.rd.bw
KO-ZNF143_mm10.bowtie2.rd.bw
WT-ZNF143_mm10.bowtie2.rd.bw
WT-H3K27me3_mm10.bowtie2.rd.bw
T_KO_1.bw
T_KO_2.bw
T_KO_3.bw
T_WT_1.bw
T_WT_2.bw
T_WT_3.bw
WT-CTCF-ckit_mm10.bowtie2.rd.bw
WT-H3K27ac-ckit_mm10.bowtie2.rd.bw
WT_input_mm10.bowtie2.rd.bw
KO-CTCF-ckit_2_mm10.bowtie2.rd.bw
KO-H3K27ac-1ckit_2_mm10.bowtie2.rd.bw
KO-H3K27me3-ckit_2_mm10.bowtie2.rd.bw
WT-CTCF-ckit_2_mm10.bowtie2.rd.bw
WT-H3K27ac-ckit_2_mm10.bowtie2.rd.bw
WT-H3K27me3-ckit_2_mm10.bowtie2.rd.bw
WT-ZNF143-ckit_2_mm10.bowtie2.rd.bw
WT_inter_30.hic
KO_inter_30.hic
ZNF143_HSLAM_st_DESeq2_FPKM.txt

Genome browser session
(e.g. UCSC)

http://137.132.97.62/public_hubs/qiling/ZNF-CTCF/hubs.txt

Methodology

Replicates

all ChIP-seq except KO ZNF143 were performed in two biological replicate. All ChIP-seq data are derived from the analysis of both replicates. Correlation of replicates was assessed with the deepTools command “multiBigwigSummary BED-file”.

Sequencing depth

KO-CTCF-ckit 23928909 16526476 76 paired-end
KO-H3K27ac-ckit 23925931 20948935 76 paired-end
KO-ZNF143-ckit 28328887 19144574 76 paired-end
KO-H3K27me3-ckit 30943700 23823431 76 paired-end
WT-ZNF143-ckit 19554128 13418185 76 paired-end
WT-H3K27me3-ckit 25232443 19519736 76 paired-end
WT-CTCF-ckit 30453013 20073152 76 paired-end
WT-H3K27ac-ckit 28628302 22768973 6 paired-end
KO-CTCF-ckit_2 32120617 21974627 76 paired-end
KO-H3K27ac-ckit_2 22747152 19332011 76 paired-end
KO-H3K27me3-ckit_2 21183615 6001007 76 paired-end
WT-ZNF143-ckit_2 20316305 13742621 76 paired-end
WT-CTCF-ckit_2 23914632 16751762 76 paired-end
WT-H3K27ac-ckit_2 23023797 19567050 76 paired-end
WT-H3K27me3-ckit_2 33205363 21968013 76 paired-end

Antibodies

Anti-ZNF143 rabbit serum generated in our lab
Anti-CTCF antibody Cat# 07-729 Millipore Lot. 2757161
Tri-Methyl-Histone H3 (Lys27) Cat# 9733BC Cell Signaling Technology Lot. 8 Clone: C36B11
Rabbit polyclonal to Histone H3 (acetyl K27) - ChIP Grade Cat# ab4729 Abcam Lot. GR261979-1

Peak calling parameters

Samples were aligned to the mouse genome (build mm10, GRCm38) with bowtie2 (v2.2.9) with default parameters. Next, non-duplicate reads that mapped to the reference chromosomes were retained using Samtools (v1.9). Peaks were identified with MACS2 (v2.1.1.20160309) for narrow peaks with the parameters ”--q 0.01--call-summits” and for broad peaks with the parameters ”--broad-cutoff 0.01”.

Data quality

sample > 5FC > -log10q(0.05)
KO-CTCF 5430 5481
KO-H3K27me3 3150 41338
KO-H3K27ac 17775 34924
WT-ZNF143 7126 9326
WT-CTCF 25622 31997
WT-H3K27ac 17178 31846
WT-H3K27me3 5813 22855

Software	Fastqc (v0.11.5), TrimGalore (v0.6.0), bowtie2 (v2.2.9), Samtools (v1.9), MACS2 (v2.1.1.20160309), HOMER (v4.10.3), bedtools (v2.25.0), bedGraphToBigWig (v4), IGV (v2.3.74), deeptools (v3.1.1), ngsplot (v2.61)

Flow Cytometry

Plots

Confirm that:

☒ The axis labels state the marker and fluorochrome used (e.g. CD4-FITC).

☒ The axis scales are clearly visible. Include numbers along axes only for bottom left plot of group (a 'group' is an analysis of identical markers).

☒ All plots are contour plots with outliers or pseudocolor plots.

☒ A numerical value for number of cells or percentage (with statistics) is provided.

Methodology

Sample preparation	After CO2 euthanasia, mice were dissected for vertebrae, femur, tibia, and hip collection. Bones were crushed using a pestle with ice cold PBS and then bone marrow cells suspension were filtered through a 70 µm BD cell strainer. Red blood cells were removed with RBC lysis buffer treatment. White blood cells were stained as described in the methods session.
Instrument	BD FACSAriaI sorter
Software	Data collection was done with BD FACSDiva software (v8.0.1) for the flow cytometry experiment.<br>Data processing was done with FlowJo (v7.5)
Cell population abundance	HSLAM: 0.005%~0.01%; ckit: 1%~2%
Gating strategy	lineage (CD3+ CD8+ B220+ CD19+ Ter119+), LSK (lineage- Sca-1+ ckit+), LK (lineage- Sca-1- ckit+), ckit (lineage- ckit+), HSLAM(LSK, CD150+ CD48-), LT-HSC (LSK CD34- Flk2-), ST-HSC (LSK CD34+ Flk2-), MPP (LSK CD34+ Flk2+), CMP (LK CD34+ FcyR-), GMP (LK CD34+ FcyR+), and MEP (LK CD34- FcyR-)

☒ Tick this box to confirm that a figure exemplifying the gating strategy is provided in the Supplementary Information.
